# Prognostic Indicators of Surgery for Esophageal Cancer: A 5 Year Experience

**DOI:** 10.4103/1319-3767.70607

**Published:** 2010-10

**Authors:** Nadim Khan, Adil Bangash, Muzaffaruddin Sadiq

**Affiliations:** Department of Surgery, Lady Reading Hospital, Post Graduate Medical Institute, Peshawar, N.W.F.P, Pakistan

**Keywords:** Albumin, esophagectomy, pre-albumin, prognosis

## Abstract

**Background/Aim:**

To assess the prognostic indicators preoperatively presenting and influencing the mortality rate following esophagectomy for esophageal cancer.

**Materials and Methods:**

This study was a retrospective cohort study, conducted at the Department of Surgery, Lady Reading Hospital, Peshawar, from 1 January 2003 till 31 December 2008. Group 1 included patients who had undergone sub-total esophagectomy and were alive at completion of 12 months; whereas Group 2 included those patients who died by the completion of 12 months. Data were recollected from the Data Bank. A list of variables common to all patients from both groups was categorized and subsequently all data related to each individual patient were placed and analyzed on the version 13.0 of SPSS^R^ for Windows.

**Results:**

Significant findings of a lower mean level of serum albumin from Group 2 were observed, whereas serum transferrin levels, also found lower in Group 2, were not statistically significant. Findings of serum pre-albumin, with a mean value of 16.12 mg/dl (*P*<0.05) and Geansler’s index for the evaluation of the presence of obstructive pulmonary disease prior to surgery showed a lower reading of mean ratio in Group 2. Anastamotic leak was not a common finding in the entire study. In most cases, the choice of conduit was the remodeled stomach. Nine patients from Group 2 were observed with evident leak on the fifth to seventh post-operative day following contrast swallow studies. This was statistically insignificant (*P* = 0.051) on multivariate analysis.

**Conclusion:**

Pre-operative variables including weight loss, low serum albumin and pre-albumin, Geansler’s index, postoperative chylothorax, pleural effusion, and hospital stay, are predictive of mortality in patients who undergo esophagectomy for esophageal cancer.

Over the past decade there have been many significant changes in the management of esophageal and gastric cancer. Both diseases have shown remarkable changes in epidemiology with a concentration of tumors adjacent to the esophagogastric junction. Keeping up with the evolving trends and evolution in the disease history itself, growing concerns over the impact of surgery on the outcome have long been debated.[1,2] Being a disease of the older age, advances in established investigative techniques and developments in new technology have radically altered the way in which the disease can be assessed without the need for surgery.[3]

With loads of literature streaming, there has been growing concern over the rising incidence of adenocarcinoma of the esophagus principally in the middle to lower thirds of the esophagus.[4,5] The increase in number of cases resistant to radiotherapy-alone therapy due to the dynamism of the disease, the preoperative patient selection for esophagogastrectomy is a field of concern. Several predictors of outcome defined preoperatively have been described in various literatures.[6,7] Keeping into consideration the frail age of most patients with the disease, comprehensive preoperative evaluation and assessment of the patient are mandatory before assigning the patient to a particular therapeutic option. Even more so the growing concern of increase in the number of patients undergoing such major procedures is undoubtedly a load on the national health care system, especially in this part of the world.[8,9]

There are few studies that have focused on the physiological states of the individual patients that affect the outcome of the disease following surgery. The increase in weight loss following surgery was remarkable, as per several studies.[10–15] Risk factors predicting the outcome of longstanding morbidity and mortality, defined by characteristics of the individual patients, need to be assessed.[16]

The role of serum proteins such as albumin, pre-albumin and transferrin has at times revealed convincing results.[17–21] Pre-operative Gaensler’s index, weight loss, and tumor location have all been implicated in the post-operative outcome of the disease. The choice of procedure performed was strictly influenced by the location of tumor, the level of lymphadenectomy intended, experience of the surgeon and stage of tumor.[22]

Following a long-term debate, transhiatal esophagectomy has been found to be associated with fewer postoperative pulmonary complications and subsequently long-term benefit in patients with resectable esophageal cancer.[23,24] The results of surgical resection for both early stage squamous cell and adenocarcinoma can be excellent. A five year survival rate is seen in over 80% of patients when tumors are confined to the mucosa and in between 50% and 80% patients when the submucosa is involved.[25,26] On the other hand, surgery plays no role in hematogenous spread in either histological variant of esophageal cancer. Preoperative risk analysis has been shown to cause a reduction in postoperative mortality from 9.4% to 1.6%.[27]

The aim of this study was to identify the pre-operative risk factors and the peri-operative impact on the outcome following esophagectomy in resectable esophageal cancers.

## MATERIAL AND METHODS

This study was a retrospective cohort study, conducted at the Department of Surgery, Post Graduate Medical Institute, Lady Reading Hospital, Peshawar, from 1^st^ January 2003 till 31^st^ December 2008. Data concerning the number of esophagectomies performed in the department over the described period were analyzed retrospectively.

Two hundred and eighty-four patients were assessed by dividing them into two groups. Group 1 included patients who had undergone sub-total esophagectomy in the described period and were alive at completion of 12 months, whereas Group 2 included the patients who had undergone sub-total esophagectomy and died by the completion of 12 months.

The inclusion criterion for patients of both groups was having a clinical diagnosis of esophageal cancer that following assessment was considered amenable to curative resection that had undergone sub-total esophagectomy. All the patients included were in the age range of 16-80 years. Any patient whose data during the evaluation period was incomplete was excluded from the study. Data was recollected from the Data Bank at Lady Reading Hospital, Peshawar. Permission from the ethical committee and related authorities was obtained, with confidentiality of both the patients and concerned staff maintained.

A list of variables common to all patients from both groups was categorized and subsequently all data related to each individual patient were placed and analyzed on the version 13.0 of SPSS^R^ for Windows. Operational definition of the common variables was designed.

Preoperative weight loss was defined as a loss of >10% bodyweight over a period of 6 months. Similarly, staging of tumours was done in concordance with the TNM classification.[28] Extravasation of radio-opaque dye at the level of anastamosis on contrast swallow conducted on the fifth to seventh post-operative day following subtotal esophagectomy was defined as anastamotic leak. Chylothorax was defined as the presence of white to creamy colored fluid in the chest drain positive for triglycerides, greater than 10ml/kg of body weight in 24 h following fifth postoperative day. Other objective data were entered as such.

Demographic and preoperative data were assessed for determining the mean and other descriptive statistics. Categorical data were compared between the groups using the χ^2^ test and the Fisher’s exact test. A *P* value of less than 0.05 was considered to be significant. Continuous variables were compared using Student’s t test. Statistical package for social sciences (*SPSS, version 13.0*; Chicago, IL, USA) was used for data analysis.

## RESULTS

Among all 284 patients who underwent esophagectomy, the perioperative deaths recorded were 11 (3.8%). Patients from both Group 1 and Group 2 had no significant differences in age (expressed in years) and male to female gender ratio. Pre-operative weight loss was significantly higher in a greater proportion of patients who underwent esophagectomy not surviving by the end of first post-operative year (Group 2). Patients from neither group had statistically significant differences in the number of patients known to have preoperative existing pulmonary disease or coronary heart disease (*P*>0.05). The mean pre-operative weight from Group 1 was greater than the mean value for Group 2 and this was also statistically significant.

Preoperative nutritional status was monitored by serial levels of serum proteins. Values denoted were obtained prior to allocation of nutrition for hyperalimentation (if given). Significant findings of a lower mean level of serum albumin from Group 2 were observed. Findings similar to serum albumin were found to be lower in Group 2 for measurements of serum pre-albumin, with a mean value of 16.12 mg/dl (*P*<0.05).

Geansler’s index for the evaluation of the presence of obstructive pulmonary disease prior to surgery showed a lower reading of mean ratio in Group 2, with a wide variation observed between individual values depicted by the standard deviation (*P*<0.05) [Table 1].

**table 1:** Pre-operative characteristics of patients undergoing esophagectomy (η=284)

Variables	Group 1 (η=185)	Group 2 (η=99)	*P* value
Age (years)	56.31 ± 11.53*	58.47 ± 9.88*	0.114^T^
Sex (M:F)	112:73	60:39	0.991χ
Pre-operative weight loss (%)	56 (30.2%)^a^	59 (59.59%)^a^	<0.05^F^
Known coronary heart disease (%)	43 (23.2%)^a^	22 (22.22%)^a^	0.845χ
Known pulmonary disease (%)	9 (4.86%)^a^	4 (4.04%)^a^	0.751χ
Pre-operative weight (kg)	60.44 ± 9.06*	57.11 ± 9.3*	0.004^T^(0.265^M^)
Serum albumin (g/dl)	3.35 ± 0.49*	2.99 ± 0.51*	<0.05^T^
Serum pre-albumin (mg/dl)	21.96 ± 6.5*	16.12 ± 5.1*	<0.05^T^
Serum transferrin (mg/dl)	204.08 ± 49*	170.3 ± 48.8*	<0.05^T^ (0.771^M^)
Gaensler index (FEV1/FVC)	79.91 ± 4.1*	77.72 ± 5.4*	<0.05^T^

Standard deviation=*;

Percentages within group=^a^;

Chi square test=^χ^;

Fisher exact test= ^F^;

Student t test= ^T^;

Multivariate analysis=^M^

Investigations revealing the characteristic of tumor were performed prior to surgery to assess for resectability. Any finding not in concert with operative findings was replaced. Majority of patients in Group1 were of stage I disease whereas patients from Group 2 were similarly divided in stage I and stage II disease; which was statistically significant. Similar findings were observed for histopathological diagnosis of tumour differentiation. The patients of Group 2 were of a higher grade (*P* <0.05).

The most common histopathological finding was adenocarcinoma found in the lower parts of the esophagus. Three patients (1.62%) from Group 1 presented with a histopathological pattern other than squamous cell carcinoma and adenocarcinoma; which were leimyosarcoma of the esophagus. Most tumors were of the lower third in both groups with the least cases arising from upper portion of the esophagus [Table 2].

**Table 2:** Tumor characteristics of patients (η=284)

Variables	Group 1 (η=185)	Group 2 (η=99)	*P* value
Stage			
I	163 (88.1)^a^	36 (36.36)^a^	<0.05^F^
II	20 (10.8)^a^	41 (41.41)^a^	
III	02 (1.08)^a^	17 (17.17)^a^	
IV	-	5 (5.05)^a^	
Grade			
G_1_	96 (51.8)^a^	29 (29.29)^a^	<0.05^F^
G_2_	86 (46.4)^a^	43 (43.43)^a^	
G_3_	03 (1.62)^a^	27 (27.27)	
Histopathology			
Squamous cell carcinoma	48 (16.8)^a^	21 (21.21)^a^	0.280χ
Adenocarcinoma	134 (47.01)^a^	78 (78.78)^a^	
Other	03 (1.62)^a^	-	
Tumor location			
Upper third	29 (15.6)^a^	14 (14.14)^a^	0.211χ
Middle third	73 (39.4)^a^	30 (30.30)^a^	
Lower third	83 (44.8)^a^	55 (55.55)^a^	

Percentages within group=^a^;

Chi square test=^χ^;

Fisher exact test= ^F^;

Figures in parentheses are in percentage

Post-operative data revealed the effect of surgical outcome for the disease that in many variables had a statistically significant effect. A fairly higher proportion of patients who had developed pleural effusion ended in Group 2. On the other hand, Group 2 also presented with a higher incidence of post-operative complications such as pneumonia and atelectasis. This would adversely affect outcomes as proved by a *P* value of 0.015, although corrected values failed to show this correlation on multivariate analysis. Anastamotic leak was not a common finding in the entire study. In most cases the choice of conduit was the remodeled stomach. Nine patients from Group 2 were observed with evident leak on the fifth to seventh post-operative day following contrast swallow studies. This was statistically significant with a *P* value of 0.008 and was also found to be non-significant on the multivariate analysis. All cases were managed conservatively. Only two patients presenting with leak died in the peri-operative period [Table 3].

**Table 3:** Postoperative data of patients (η=284)

Variables	Group 1 (η=185)	Group 2 (η=99)	*P* value
Pleural effusion	38 (20.5%)^a^	34 (34.34%)^a^	0.011χ
Pulmonary complications	39 (21.08%)^a^	34 (34.34%)^a^	0.015χ (0.089 M)
Anastamotic leak	04 (2.16%)^a^	09 (9.09%)^a^	0.008χ (0.051^M^)
Hoarseness	10 (5.4%)^a^	-	0.019χ (0.066^M^)
Chylothorax	02 (1.08%)^a^	05 (5.05%)^a^	0.04χ
Surgical procedure			
Transhiatal esophagectomy	99 (53.5%)^a^	62 (62.62%)^a^	0.140χ
Transthoracic esophagectomy	86 (46.4%)^a^	37 (37.37%)^a^	
Hospital stay (days)	7.71 ± 2.16*	7.93 ± 2.64*	0.39^M^

Standard deviation=*;

Percentages within group=^a^;

Chi square test=^χ^;

Student t test= ^T^;

Multivariate analysis=^M^

Chylothorax was observed in five cases (5.05%) from Group 2 as compared to Group 1 where two patients developed chylothorax (0.04). One case of post-operative chylothorax formation required re-exploration as the triglyceride rich fluid in the chest drain failed to remit following conservative management.

The choice of procedure being the surgeon’s decision depending on expertise, personal preference, and location of the tumor did not significantly alter the distribution of patients into respective groups (*P*=0.140). However, a greater number of tranhiatal esophagectomies were performed during the entire study period. No significant difference was observed in the length of hospital stay in both groups; with an average hospital stay of 7.71 ± 2.16 days and 7.93 ± 2.64 days in Group 1 and Group 2, respectively (*P*=0.439), but this value greatly changed as being significant on the multivariate study (*P*=0.39).

Unfortunately, the study at hand was a retrospective analysis of the data evaluated over a 5 year duration. Thus, the study failed to target a specific subset of the population diagnosed with esophageal carcinoma. A great variation existed between both groups. It was thus recommended that a multivariate analysis with tests of between subject effects be applied. The major confounders in depicting the significant differences between the two groups were the histology and the stage of the tumor. No significant difference was thus observed for age and gender with a *P* value >0.05. Although a significant prognostic effect of pre-operative weight on the outcome following surgery was observed, this failed to suffice to the multivariate studies conducted and sum of III squares for the effect with histology and stage failed to show any significance (*P*=0.265).

The effect of pre-operative weight loss, pre-operative albumin, pre-albumin, and Gaensler’s index all showed significance on multivariate analysis. The exception was to the effect that had been observed for patients with a low transferring level between the two groups. The *P* value found for this variable was 0.771. The analysis was also extended to the other variables in the post-operative category that were found to be significant by application of the chi-square test.

The relation of post-operative pulmonary complications greatly differed on further analysis. This variable failed to show a correlation (*P*>0.05) and so was the effect observed with the incidence of post-operative leak from the anastamotic site (*P*=0.51). Among the post-operative complications only chylothorax and pleural effusion demonstrated an effect over the outcome and subsequent placement of cases into either group.

## DISCUSSION

The epidemiological characteristics of the disease shown from this study with respect to tumor characteristics show the changing patterns and evolution of the history of the disease.[29] With a swing from higher number of squamous cell carcinoma to adenocarcinoma being the more frequent form on histological grounds and more importantly the shift of location to a more distal level of esophagogastric region; this study has proved its worth.[30]

The outcome following a major undertaking for a grave disease such as esophageal cancer has greatly altered in the past few decades due to improvements in post-operative care and modifications in treatment protocols.[31] There are not many studies done to comment on the effect of preoperative status where a convincing guideline to the selection of a particular procedure be allocated to a group of patients with esophageal cancer.[32] The consideration is that the balance be focused to patient benefit. Some studies recommend the major procedure as a palliative measure for the relief of dysphagia in esophageal cancer not amenable to a complete clearance.[33]

In this study, patients were grouped on the basis of 1 year mortality figures. Debate to the short-term outcome following esophagectomy for esophageal carcinoma has been extensively explored.[34–36] To date few studies from this region have focused on the patient characteristics and long-term benefits of surgery. Certainly, the post-operative morbidity related to the operative technique could not be emphasized more. Due to the retrospective nature of this study, the listed variables are far fewer than would be anticipated.

The association of a higher incidence of mortality following esophagectomy in patients presenting in the pre-operative period with weight loss has been outlined previously by Žurauskas *et al*.[37] Keeping in focus the statistical significance of this association, the high proportion of weight loss history prior to surgery in Group 1 patients is over-ruled. Whether the notion that this finding is in association with the lack of reserve to withstand the trauma generated by the major procedure or implicating the pre-operative nutritional status is debatable.

Pre-operative nutritional status and its relation to the levels of serum albumin, pre-albumin, and transferrin were another challenge in this study.[38,39] There was a higher incidence of mortality with lower pre-operative values of albumin and prealbumin in the serum.[40] Insignificant association was seen with serum transferrin levels with the mean serum transferrin levels lower in Group 2. The role of hyperalimentation that was instituted to some patients included in the study; to avoid the confounding association, the serum proteins levels analyzed were values obtained before nutritional modifications.

The stage of the tumor from both groups showed a higher proportion of patients from Group 2 with later stage disease that may have confounded other post-operative variables. Similar was the case with the number of cases in Group 2 that presented with tumors of a higher grade. However, the location of tumor and results of tumor histology did not adversely confound the outcome of post-operative variables, as the difference between both groups was not significant.

The incidence of postoperative morbidity had strong statistical significance on the 1 year mortality rate. The incidence of leak was significantly associated with mortality at the end of 1 year yet on multivariate studies, this failed to be demonstrated.[41–42] Most of these cases were yet managed conservatively. The incidence of postoperative pleural effusion was higher in Group 2 and was statistically significant (*P*=0.011). Pleural effusion is a result of a faulty technique during transhiatal esophagectomy as the pleural cavity is entered.[43] A greater proportion of postoperative pulmonary complications and chylothorax was also observed in Group 2 which were significant.[44]

The debate to which patient is most benefited in the long run from the effect of curative surgery is a matter of concern because of the recent advancement in procedures undertaken to palliate. Laser can be performed repeatedly and innumerably in some case with earlier and longer durations of remission from dysphagia.[45] Similar are the results, yet variable, with photocoagulation, argon beam therapy, and esophageal stenting.[46–48] The role of surgery in the palliative relief of dysphagia in patients with esophageal carcinoma was not assessed in this study. The choice of conduit was not part of analysis in this study.

The criteria for selection of patients varies greatly from centre to centre and from surgeon to surgeon. The need for more comprehensive yet accurate mode of patient selection is required. The role of pre-operative co-morbid conditions such as chronic obstructive pulmonary disease and coronary heart disease in the surgical management of esophageal cancer has been discussed. More so variables like Geansler’s index has been tested and as in this study adversely affects the outcome. The role of peri-operative parenteral nutrition monitored by the level of serum proteins like serum albumin and pre-albumin has been noted in this study. In this case, there was strong association of mortality with the levels of serum proteins. Whether the requisition of serum albumin prior to surgery has a definitive role in improving outcome for patients undergoing esophagectomy in malignant disease has yet to be validated.

## CONCLUSION

Pre-operative variables including weight loss, low serum albumin and pre-albumin, Geansler’s index, all have strong predictive relation to mortality on patients who undergo esophagectomy for esophageal cancer. Post-operative morbidity such as pleural effusion and chylothorax following esophagectomy all increase the mortality rate. The need for a comprehensive criterion of patient selection for curative esophagectomy with resectable esophageal carcinoma is required.
